# Mechanism of CD38 via NAD^+^ in the Development of Non-alcoholic Fatty Liver Disease

**DOI:** 10.7150/ijms.81381

**Published:** 2023-01-22

**Authors:** Min Dong, Shuo Wang, Zuowei Pei

**Affiliations:** 1 Department of Cardiology, Beijing Hospital, National Center of Gerontology, Institute of Geriatric Medicine, Chinese Academy of Medical Sciences, Beijing, China.; 2 Department of Internal Medicine, The Affiliated Zhong Shan Hospital of Dalian University, Dalian, China.; 3 Department of Cardiology, Central Hospital of Dalian University of Technology, Dalian, China.; 4 Faculty of Medicine, Dalian University of Technology, Dalian, China.

**Keywords:** Non-alcoholic fatty liver disease, CD38, NAD^+^, Macrophage-1 Insulin resistance, Lipid accumulation.

## Abstract

Non-alcoholic fatty liver disease (NAFLD) is the most common chronic liver disease globally, and it can proceed to cirrhosis and hepatocellular carcinoma, as well as cardiovascular disease, chronic renal disease, and other complications, resulting in a massive economic burden. At the moment, nicotinamide adenine dinucleotide (NAD^+^) is thought to be a possible treatment target for NAFLD, besides Cluster of differentiation 38(CD38) is the primary NAD^+^ degrading enzyme in mammals and may play a role in the pathophysiology of NAFLD. For example, CD38 regulates Sirtuin 1 activity and hence affects inflammatory responses. CD38 inhibitors enhance glucose intolerance and insulin resistance in mice and lipid accumulation in the liver is greatly decreased in CD38-deficient mice. This review describes the role of CD38 in the development of NAFLD in terms of Macrophage-1, insulin resistance, and abnormal lipid accumulation in order to offer recommendations for future NAFLD pharmacological trials.

## Introduction

Non-alcoholic fatty liver disease (NAFLD) is the most prevalent kind of chronic liver disease in the world, accounting for 25.24% of all cases, and is rapidly becoming the primary cause of liver-related morbidity and mortality 1. NAFLD encompasses a range of pathological changes including non-alcoholic fatty liver (NAFL) and nonalcoholic steatohepatitis (NASH), and can progress to cirrhosis and hepatocellular carcinoma (HCC) 2. NAFLD can also cause extrahepatic symptoms such as cardiovascular disease chronic renal disease, and respiratory illness 3. However, the pathogenesis of NAFLD is unknown. Nicotinamide adenine dinucleotide (NAD^+^) has been identified as a potential target for NAFLD prevention and reversal. NAD^+^ is a critical cofactor in redox processes and a key regulator of several mammalian metabolisms. It is involved in a variety of biological processes and is essential for energy production, fatty acid and cholesterol synthesis, oxidation reactions, adenosine triphosphate (ATP) production, gluconeogenesis and ketogenesis 1, 4. The cluster of differentiation 38(CD38) is a single-chain type II transmembrane glycoprotein with a large extracellular domain at the C-terminus and a small cytoplasmic domain at the N-terminus 5. It was first identified as a lymphocyte-specific antigen in lymphocytes, but it was soon discovered to also be present intracellularly, where it is responsible for metabolizing intracellular NAD^+^ and generating second messengers like cyclic adenosine dinucleotide phosphate ribose (cADPR) in mammalian cells, as well as in pathological conditions that promote the development of a variety of diseases like cardiovascular disease, aging, obesity, diabetes, and inflammation. 5, 6. CD38 is one of the major NAD glycohydrolase (NADase) in mammals, and it is currently believed that NAD^+^ levels can be effectively increased by inhibiting CD38, several inhibitors of CD38 exist or are under development 7-9. As of now, dietary and lifestyle modifications are the cornerstone of treatment for NAFLD and NASH despite the fact that there is no Food or Drug Administration-approved drug for these conditions. However, the sustainability of these treatments over the long term is poor. This review covers the role of CD38 on NAD^+^ and explores the effect of CD38 on the onset of NAFLD to clarify the pathophysiology of NAFLD further and offer ideas for clinical therapeutic research.

### Typing and function of CD38

A cADPR synthetase is called CD38. The cADPR synthase indirectly influences a number of Ca^2+^-dependent processes, such as cell proliferation, muscular contraction, immunological response, and glucose-stimulated insulin production by pancreatic β-cells 10. It has been demonstrated that flavonoids, such as apigenin and quercetin, decrease CD38 activity. These flavonoids raise the level of NAD^+^ in hepatocytes and may attenuate NAFLD 11. According to studies, CD38 is a membrane protein with an extracellular type II and an intracellular type III orientation 12. Extracellular NAD^+^ is hydrolyzed to form nicotinamide and ADP-ribose at the catalytic site, which is directed toward the extracellular space (type II CD38). The extracellular enzymatic properties of type II CD38 raised long ago the question about the topological paradox of the entry of intracellular NAD^+^ substrates into the extracellular active site and the entry of extracellular cADPR products into its intracellular receptor ryanodine (RyR) channel, besides, according to the topological paradox theory, intracellular cADPR can be generated 13, 14. The salvage pathways then utilize nicotinamide for the production of NAD^+^ 5. It was shown that cellular levels of NAD^+^ were differentially increased in the tissues of CD38-deficient mice and that CD38-deficient protected mice from high fat diet (HFD)-induced obesity^15^.It has been demonstrated that CD38 (type IIICD38) can be introduced into the endoplasmic reticulum (ER) membrane with its catalytic site facing the cytoplasm^16^.However, the type III CD38 catalytic structural domain is cytoplasm-oriented with low abundance, but can effectively cyclize cytoplasmic NAD^+^ to generate cADPR^17^. The type of membrane insertion may be determined by protein kinases. Protein kinases may diminish the charge of positive amino acids close to the transmembrane domain 16. Nicotinamide adenine dinucleotide phosphate (NADPH) oxidases have the ability to activate Type III CD38, which first appears to be dormant 18. In endolysosomal membranes, type II CD38 is thought to play a role in the production of the calcium-mobilizing second messenger nicotinic acid adenine dinucleotide phosphate (NAADP), While type III CD38 is thought to contribute to the ER membranes' production of the calcium-mobilizing cADPR^16^. There is still much to learn about the regulation, localization, and function of CD38.

NAFLD is a metabolic disease with a complex etiology involving inflammation, excessive fatty acid accumulation and insulin resistance.^2^. NAD^+^ is currently thought to be a promising target for the therapy of NAFLD. CD38 is the major depleting enzyme of NAD^+^ in mammalian tissues. Mice deficient in CD38 have increased NAD^+^ in the brain and liver, suggesting that CD38 plays a critical role in maintaining NAD^+^ homeostasis 19.As a kind of NAD^+^-consuming enzymes, CD38 may play a specific role in the development of NAFLD.

### Roles of CD38 in NAFLD

#### 1. The activation of M1 macrophages

Macrophages in the liver, also known as Kupffer cells, accounts for 15% of total hepatocytes and 80-90% of all tissue-resident macrophages. Activation of Kupffer cells is closely associated with the development of NAFLD 20, 21. It has been demonstrated that CD38 is a Macrophage-1(M1) specific gene 22. Obesity increases adipose tissue and the liver must absorb excess lipids stored in lipid droplets, leading to ongoing inflammation and activation of resident and infiltrating macrophages 11. Studies have shown that CD38 cleaves NAD^+^ inside and outside the cell and that a large fraction of CD38 ecto-NADase activity in macrophages is intracellular 23. Besides, enhanced NADase activity of pro-inflammatory M1 macrophages is mediated by CD38^23^. Sirtuin 1 (SIRT1) activation attenuated hepatic steatosis and inflammation in HFD-induced NAFLD by inhibiting CD38 expression and NF-κB signaling pathway 24-26. Moreover, CD38 deficiency has been demonstrated to shield the heart from HFD-induced oxidative stress by activating the Sirtuin3 (SIRT3)/FOXO3-mediated antioxidant stress pathway 15. However, there are no studies of this pathway in the liver. It is possible that pro- and anti-inflammatory responses seem to be related to cell type and cellular environment. Inhibition of the ectoenzymatic activity of CD38 *in vivo* with antibody 68(Ab68) leads to a decrease in the level of the NAD^+^ degradation product adenosine diphosphate ribose (ADPR) 27. It was found that inflammation can reduce NAD^+^ by increasing CD38 and that blocking the exoenzyme activity of CD38 can increase NAD^+^ through a nicotinamide mononucleotide (NMN)-dependent process 27.

Recent findings reveal a major role for CD38 in inflammation, suggesting that the age-related decline in NAD^+^ is mediated in part by aging/ the senescence-associated secretory phenotype (SASP)-induced accumulation of CD38 inflammatory cells in tissues 23. Besides, chronic inflammation affects energy metabolism by interfering with the AMPK-NAD^+^-PGC1α-SIRT1 pathway via CD38^28^. This may provide new clues for further studies to explore the exact mechanism of CD38 in the progression of NAFLD to cirrhosis 23, 27. Hepatocytes with NAFLD have considerably increased glycolysis, which raises the amounts of pyruvate in the blood and liver. The precise mechanism by which increased glycolysis can result in hepatic inflammation is unknown 29. Clinical research on the interactions between various organs in patients will ultimately be important. Future research must take into account novel players in NAFLD and lipid homeostasis, such as lipid droplet dysregulation. NAFLD is linked to mutations in the lipid droplet lipase PNPLA3 (patatin-like phospholipase A3) 1, 30. It was discovered that PTIP, also known as Paxip1 (interaction of Pax with transcriptional activation domain protein-1), controls the expression of CD38 in macrophages by creating an H3K27ac-enriched enhancer of CD38 activity in conjunction with acetyltransferase p300.Additionally, by comparing histone changes with gene expression profiles for NAD^+^ metabolism, PTIP was discovered to be a crucial regulator of CD38 expression, an enzyme that predominantly uses NAD^+^ in macrophages 31.

#### 2. Insulin resistance

Type 2 diabetes (T2DM) and insulin resistance are both directly related to the evolution of NAFLD 32. Besides, improved NASH histopathology and fibrosis regression were both strongly correlated with enhanced insulin sensitivity 33. The largest independent risk factor for the onset of cirrhosis and HCC in NASH patients was a baseline diagnosis of T2DM 33, 34. A recent study has demonstrated that the decline of NAD^+^ levels with aging are primarily dependent on CD38, besides, CD38 deficiency activates the NAD^+^/SIRT1 pathway 35, 36. Activation of SIRT1 improves glucose tolerance, lowers hyperinsulinemia, and increases systemic insulin sensitivity by significantly repressing the jun-n-terminal kinase (JNK) and inhibitor of NF-κB kinase (IKK) inflammatory pathways 37. CD38-deficient mice exhibited improved glucose tolerance compared to wild-type mice 35. 78c is a specific inhibitor of CD38 and does not directly affect the activity or expression of other enzymes involved in NAD^+^ metabolism, it could improve glucose intolerance and insulin resistance in mice 8. Insulin resistance promotes adipose tissue lipolysis, resulting in elevated levels of non-esterified fatty acids (NEFA) in the blood, which are taken up by the liver. Serum insulin levels were greater in CD38-transgenic mice than in control mice in a glucose tolerance test performed on live animals 38. When anti-CD38 antibodies are present in patients, glucose-induced insulin secretion may be impaired and lead to type 2 diabetes 38. The researchers demonstrated that peroxisome proliferators-activated receptor γ(PPARγ)-mediated insulin sensitization enhances NAADP production by upregulating CD38 in adipocytes and that CD38^-/ -^completely blocked this effect 36. Cyclic ADP-ribose acts as a second messenger for intracellular Ca^2+^ mobilization in glucose-induced insulin secretion 39. This suggests that CD38 plays a key role in enhancing insulin sensitivity. However, more experiments are needed to explore the mechanism of CD38's role in the relationship between NAFLD and insulin resistance.

#### 3. Lipid accumulation

One of the critical pathogenic characteristics of NAFLD is excessive lipid accumulation 2. It has been demonstrated that CD38-deficient animals have much less lipid accumulation in their livers 40. It is possible that the energy depletion is caused by increased NAD^+^ due to CD38 deficiency and the data suggest that levels of NAD^+^ and the NAD^+^ precursor nicotinamide mononucleotide (NMN) are significantly increased 40. CD38 inhibition in a mouse model of HFD-induced obesity treated with the flavonoid apigenin showed a reduction in lipid accumulation in the liver through increased lipid oxidation 41. Additionally, elevated CD38 expression in cells causes mitochondrial metabolic dysfunction 35. Part of the mechanism may be increased lipid oxidation, NAD^+^ reduction and SIRT1 activation 36, 41. The exact mechanism requires further research. In the HFD model, CD38-deficient mice displayed considerably less hepatic fat infiltration than wild-type controls 42. However, more data are needed to demonstrate that CD38-deficient mice can prevent the abnormal accumulation of hepatic lipids in NAFLD. CD38 is also associated with the browning of white fat and the development of "classic" brown fat in mice. NAD^+^ and NADP(H) levels in thermogenic adipose tissues are increased when CD38 is downregulated 43. Ablation of SIRT3 in CD38-deficient mice eliminates the protective role of CD38 inhibition in HFD-induced obesity 35. Thus, the role of CD38 as a regulator of obesity and energy expenditure may also be related to thermogenesis and mediated by a NAD^+^-SIRT-dependent mechanism. Increased glutathione/oxidative glutathione (GSH/ GSSG) ratio in CD38-deficient hearts was found to be one of the mechanisms that reduce HFD-induced oxidative stress. Progress studies are needed regarding the mechanism of oxidative stress in the liver by CD38^15^. In pre-diabetic patients, CD38 cells were negatively correlated with cholesterol and low-density lipoprotein, and CD38 cells were negatively correlated with high-density lipoprotein values in patients with T2DM 44. The study demonstrated that CD38-deficient mice were substantially more resistant to the obesity-inducing effects of HFD, and that WT mice fed HFD had significantly higher CD38 expression 36. This protective mechanism may be related to the inhibition of PPARγ and CCAAT/enhancer-binding proteinα (C/EBPα) expression in adipose tissue 36. The expression and activity of sterol regulatory element-binding protein-1(SREBP1) and its target gene fatty acid synthase (FASN) were found to be significantly diminished in CD38^-/ -^ mouse embryonic fibroblasts (MEFs) 36. This suggests that CD38 deficiency not only inhibits adipocyte differentiation, but also suppresses adipogenesis.

## Conclusion

NAFLD is the most common form of liver disease, but there are no licensed medications available for its treatment. The progression of NAFLD is closely related to chronic inflammation, insulin resistance, and lipid accumulation. NAD^+^ is currently considered as a potential therapeutic target for NAFLD. As the critical NAD^+^ degrading enzyme in mammals, inhibiting of CD38 can significantly increase NAD^+^ levels and prevent NAFLD progression. These ideas have been confirmed in animal studies, but clinical trials still need to be verified.

## Outlook

In summary, there is still a long way to go in the pathogenesis and clinical studies of CD38 in NAFLD. First, the pathogenesis of CD38 in NAFLD has not been adequately studied, and more studies are needed to demonstrate the correlation between CD38 and inflammation, abnormal accumulation of fatty acids, and insulin resistance. The interaction of inflammation and abnormal lipid accumulation promotes the pathological development of NAFLD, while abnormal lipid accumulation can be caused by insulin resistance. Abnormal accumulation of lipids in the liver leads to chronic inflammation and activation of Macrophage-1, and the development of inflammation in turn acts on hepatocytes, leading to abnormal accumulation of lipids. This interaction complicates the study of the pathogenesis of NAFLD, so more experimental investigations are needed in the future.

## Figures and Tables

**Figure 1:**
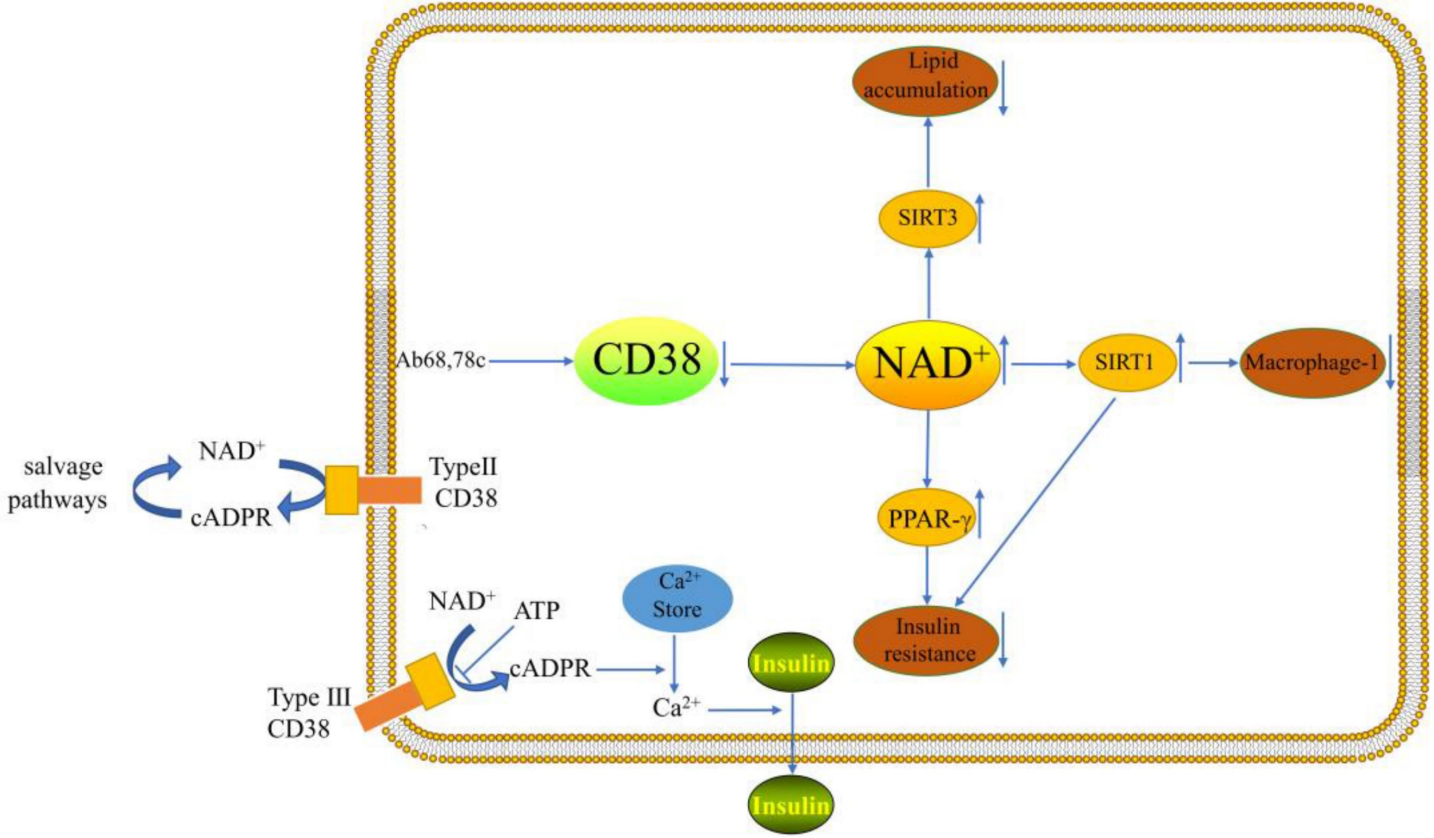
CD38 can influence the course of NAFLD through lipid accumulation, macrophage-1 and insulin secretion.
